# Quasi-Static Penetration Properties of 3D-Printed Composite Plates

**DOI:** 10.3390/ma17112536

**Published:** 2024-05-24

**Authors:** Axel Baruscotti, Yuri Borgianni, Franco Concli

**Affiliations:** Faculty of Engineering, Free University of Bolzano, 39100 Bolzano, Italy

**Keywords:** quasi-static indentation test, composite plate, Fused-Filament-Fabrication, continuous fibers, short fibers, impact, Additive Manufacturing

## Abstract

This work investigated the impact and piercing load resistance (energy absorption capabilities) of 3D-printed composites plates manufactured by means of the Fused-Filament-Fabrication (FFF) technique. Two sets of reinforced composite plates were produced. The first set of plates was printed with short-carbon-fiber-reinforced polyamide-12, while the second set was reinforced with continuous fibers. The plates were tested with quasi-static indentation tests at various Span-to-Punch ratios and with three different indenter nose shapes (blunt, hemispherical, and conical). The quasi-static measurements were subsequently elaborated to estimate the energy absorption capability of the plates during a ballistic impact. The addition of continuous fibers increased the quasi-static energy absorption capability by 20–185% with respect to the short-fiber-reinforced plates. The quasi-static results showed that by including the continuous reinforcement in the plates, the normalized energy absorbed increased by an order of magnitude. Finally, a comparison with data from the literature concerning continuous-reinforced composite plates manufactured by means of traditional techniques was carried out. The comparison revealed that FFF-printed composite plates can compete with traditional composite ones in terms of both ballistic and quasi-static penetrating load conditions, even if limited by the lower fiber volume fraction. Thus, these findings confirm that this novel Additive Manufacturing technique is promising and worth investigating further.

## 1. Introduction

The study of the mechanical properties of composite materials produced by means of the Fused-Filament-Fabrication (FFF) process is a relatively new field of research, particularly studies involving continuous fibers as reinforcement. The first paper on this topic was published by Matsuzaki et al. in 2016 [1]. The promise of this novel Additive Manufokacturing (AM) process is to enable the production of functional structural parts, thus overcoming the limitations of traditional polymeric non-reinforced components produced with FFF. Starting from 2016, a growing interest in this research branch was observed, and more and more studies involving the mechanical characterization of the FFF-printed composites have been published in the scientific literature. However, the characterization has mainly been limited to quasi-static tensile, bending, shear, and compressive behaviors of the printed specimens, while only a limited number of studies have focused on the impact behavior, as shown by Safari et al. [2] in their review. Even in the last 2–3 years, the number of publications involving the evaluation of the impact performance of FFF-printed composites reinforced with continuous fibers is very limited, which highlights a substantial lack of literature compared to the domain of composite materials produced through traditional processes. In other words, it seems that a comprehensive evaluation of the impact performances of FFF-printed composite plates has not been performed yet. Most of the studies focus on the influence that the single printing process’ variables, such as materials, layer stacking, reinforcement orientation, etc., have on the impact performance of the printed part, ref. [3], rather than trying to thoroughly evaluate its overall impact resistance in terms of energy absorption capability. For this purpose, quasi-static punch tests are commonly employed. Wang et al. [4] focused their research on polyamide/continuous Kevlar/continuous carbon fiber composites. In their research, Wang et al. focused on the effect of the layer stacking sequence for thin composite plates, rather than the reinforcement layout in the single layer. The researchers were able to show that the most efficient position for the fiber-reinforced layers is the middle section of the plate, which is enclosed between a top and bottom layer of non-reinforced matrix [5]. Regarding the choice of the reinforcing fibers, Goh et al. [5] performed quasi-static punch tests on thin composite plates reinforced with either glass fibers or carbon fibers. The results showed a higher energy absorption capability in the case of the plate reinforced with glass fibers; however, it is worth noticing that the uncertainty related to the carbon fiber plate was significant. Besides quasi-static punch tests, other impact characterization techniques have increasingly been used in the last years to evaluate impact and penetration performance. In this regard, a study by Kabir et al. [6] focused on the impact characterization of glass-fiber-reinforced polyamide plates by means of drop-weight impact tests. The objective of the study was to evaluate the best-performing fiber layout, which turned out to be the orthogonal one (0°/90°), in agreement with the findings of Wang et al. The same research group led by Kabir [7] performed a similar study on the combined effect of fiber layout and infill cellular structures, showing that the main parameter influencing the impact performance of the 3D-printed composite plates is the fiber layout, again confirming that the best fiber configuration is the orthogonal one. Caminero et al. [8] explored the effect of four main parameters (reinforcing material, build orientation, layer thickness, and fiber volume fraction) on the energy absorption capabilities of 3D-printed composite specimens by means of Charpy’s impact test. The results confirmed general observations already present in the literature, such as the observation that the fiber volume fraction plays a significant role and that glass fibers are the most performant reinforcing material, followed by Kevlar and carbon fibers. Li et al. [9] studied the impact properties of 3D-printed specimens made of carbon-reinforced PA6 by means Charpy’s test. The researchers found that the orthogonal layout behaved worse compared to the unidirectional one, in disagreement with the results coming from out-of-plane impact or penetration tests, thus suggesting that Charpy’s test may not be the most suitable testing procedure to evaluate the ballistic behavior of 3D-printed composite plates [7]. Fraccaroli et al. [10] studied the dynamic behavior of a multilayer ski via numerical approaches based on quasi-static tensile tests combined with Digital Image Correlation measures [8]. Cersoli et al. [11] performed experiments on a 3D-printed continuous-fiber-reinforced composite based on a coaxial Kevlar/PLA filament. Li et al. [9] performed, in addition to the abovementioned characterization, some Charpy impact tests in which the impact strength was measured, while Cheng et al. [12] studied the quasi-static penetration property of 3D-printed woven-like ramie-fiber-reinforced bio-composite materials, namely, thin composite plates made of PLA reinforced with continuous natural fibers. The researchers evaluated the energy absorption capabilities of the printed plate for different fiber layouts and showed that the orthogonal layout (0°/90°) is significantly more efficient than the unidirectional one. Moreover, the study showed that the penetration mechanism is significantly affected by the indenter’s dimensions; smaller indenters can concentrate the load on smaller areas. Bio-composites have also been studied by Taghizadeh et al., who investigated the behavior of Multilayer Composite Laminates under Low-Velocity Impacts [13].

To thoroughly evaluate overall impact resistance in terms of energy absorption capability, quasi-static punch tests are commonly employed in the available studies, but they rarely systematically consider all of the relevant parameters, such as the Span-to-Punch ratio (SPR) [14,15] or the indenter’s nose shape [16,17], that have been shown to be critical in determining the overall impact performance of composite materials produced through traditional fabrication means. Therefore, a thorough experimental investigation on the out-of-plane loading response of FFF-printed composites considering all of the impact-relevant parameters is needed to determine the effectiveness of this novel AM process in producing structural parts that are also capable of withstanding impact and piercing.

## 2. Materials and Methods

### 2.1. 3D Printing Equipment

The Anisoprint Composer A3 from Anisoprint Inc., Rome, Italy, available at the Rapid Prototyping and Additive Manufacturing Lab of the Free University of Bolzano, was used to fabricate the composite plate specimens used in the present study. This FFF printing machine allows for additively manufacturing polymeric parts reinforced with a continuous fiber filament. The printer has two independent nozzles; the first one conveys only a polymeric filament, while the other allows for the co-extrusion of a pre-impregnated fiber filament and another polymeric filament, which work as a matrix. Regarding the materials used (see Table 1), Smooth PA is a proprietary filament based on a polyamide PA12 filled with chopped carbon fibers that is meant to be used for printing portions of the part that do not need to be reinforced or that need a better surface quality. As for the co-extrusion of the reinforced filament, instead, another proprietary nylon filament, CFC PA, was used in combination with a reinforcing filament CCF-1.5K in the form of a tow made of 1500 carbon monofilaments, which were impregnated with a special polymer, thus ensuring good interface bonding between the reinforcement and the matrix.

### 2.2. Plate Specimens

**Figure 1:**
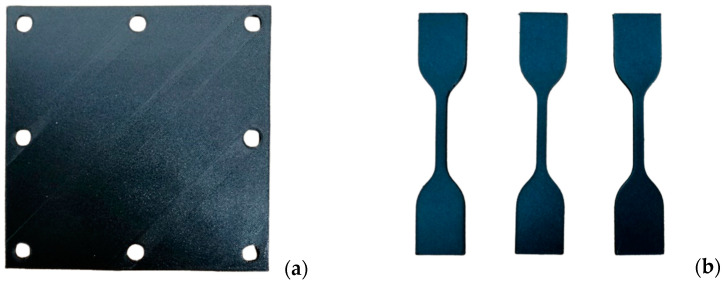
(**a**) The composite plate specimen and (**b**) the coupons for the tensile test.

The plate specimen consisted of a plate with the dimensions 150 mm × 150 mm × 3 mm, with eight perimetral holes 10 mm in diameter spaced 10 mm from the border and 65 mm from each other (see Figure 1a); this was performed to match the holes in the support plates of the testing equipment [18].

The specimens were printed with the Anisoprint Composer A3 after proper selection of the printing parameters by means of the Aura software. The selection of the printing parameters (see Table 2) was performed according to work by Liu et al. [19] with the Anisoprint Composer A3, in which the scholars investigated the influence of four main process parameters—layer thickness, hatch spacing, nozzle temperature, and printing speed—on the bending stiffness and flexural strength of PETG (polyethylene terephthalate glycol) specimens reinforced with a continuous carbon filament.

**Figure 2:**
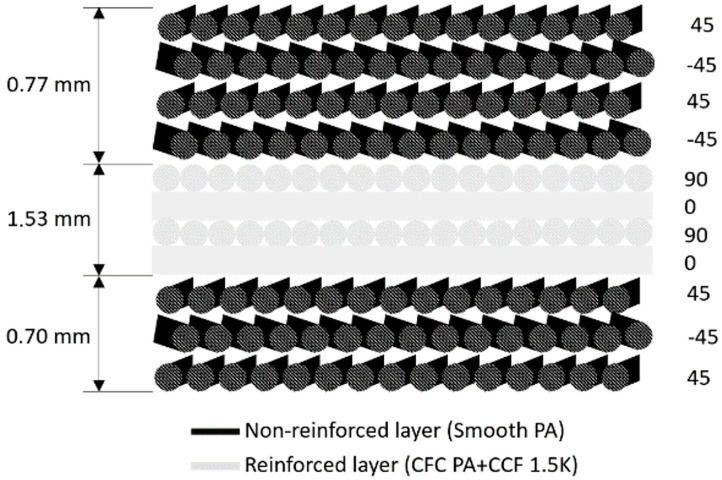
Schematic representation of the layers composing the plate reinforced with continuous fibers.

The choice of the printing parameters resulted in a plate with four layers of reinforcement enclosed by seven layers of non-reinforced material, which built up an external shell protecting the reinforced layers (see Figure 2).

The estimated fiber volume fraction (V_f_) for the specimens reinforced with continuous fibers was 23%, and the magnitude was consistent with the fiber volume fraction found in the literature for most FFF printing techniques [20]. However, it is worth pointing out that this is only an estimation, as no print flaws, such as voids or print errors, were considered. Moreover, it is noteworthy that the short fibers present in the Smooth PA were not considered in the estimation. In addition to the composite plates reinforced with the continuous fiber, which were the main object of the present study, an equivalent number of composite plates reinforced only with short fibers, i.e., Smooth PA only, were produced as a control group. This was performed to allow for the quantification of the reinforcement provided by the addition of the continuous fiber filament. However, in the case of the non-reinforced plate, the infill layout was made with a 100% infill triangular–cellular pattern. In total, 24 composite plates were produced, including 12 plates reinforced with continuous fibers and 12 plates reinforced with short fibers. The print time for each short-fiber-reinforced plate was 8.5 h, while for each of the continuous-fiber-reinforced plates, it was 10.5 h.

### 2.3. Quasi-Static Indentation Test Equipment

**Figure 3:**
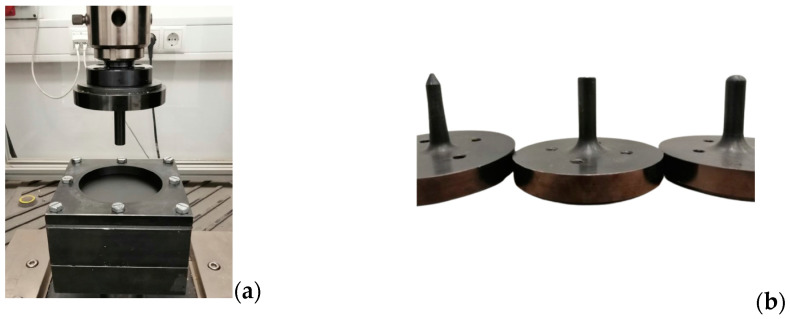
Schematic representation of (**a**) the equipment used to perform the quasi-static indentation tests and (**b**) the indenters.

To perform the quasi-static indentation tests, an MTS Criterion 100 universal testing machine (MTS Systems, Eden Prairie, MN, USA) available at the Materials Characterization Lab of the Free University of Bolzano was equipped with an indenter 12.7 mm in diameter and a clamping system made of two drilled plates to fix the specimens, as shown in Figure 3. Both of the two drilled plates and the specimen were sized 150 × 150 mm, and they were kept together by means of eight M8 bolts fastened inside the eight perimetral holes spaced 10 mm from the border and 65 mm from each other. In total, three sets of support plates, each one made of two drilled plates with different hole diameters (25.4 mm, 50.8 mm, and 101.6 mm, respectively), were used in order to obtain three different SPR values of 2, 4, and 8. Moreover, three different indenter nose shapes—blunt, hemispherical, and conical—were used for the tests. To reproduce the crushing test with SPR = 0, a lower support plate without a hole and the upper support plate for the SPR = 2 test were used, thus allowing for a testing condition as close as possible to the one leading to SPR = 0. All of the indentation tests were performed with a relative velocity of 1 mm/min to eliminate the influence of the strain rate [18].

### 2.4. Quasi-Static Penetration Model (QSPM)

Gama and Gillespie [14] proposed a quasi-static penetration model to estimate the energy absorbed by a composite plate during ballistic impact based on quasi-static punch tests with varying SPR values.

According to the model, the energy absorbed by the plate $E_{QS}^{PM}$, see Equation (1), can be obtained by considering two contributions: the energy related to the integration of the hydrostatic envelope $E_{HS}^{env}$, which can be attributed to the energy expended to damage the material, and the energy $E_{el}\;{|}_{SPR=8}^{P_{max}}$, derived from the test with SPR = 8, which represents the elastic strain energy stored in the plate just after impact.

(1) $$E_{QS}^{PM}=E_{HS}^{env}+E_{el}\;{|}_{SPR=8}^{P_{max}}$$

**Figure 4:**
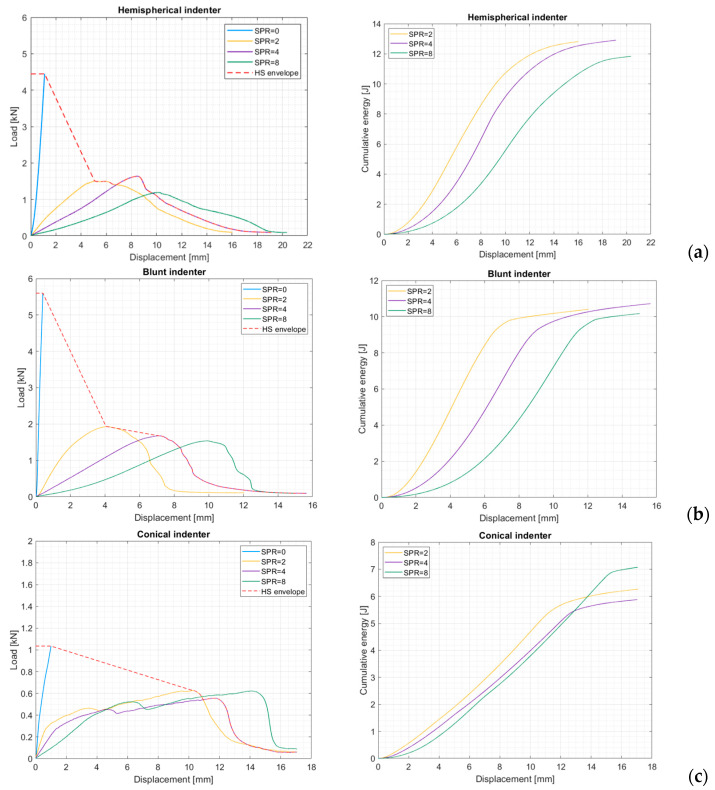
Force–displacement curves for short-fiber-reinforced composite plates and cumulative energy vs. displacements for (**a**) the hemispherical, (**b**) the blunt, and (**c**) the conical indenters.

**Figure 5:**
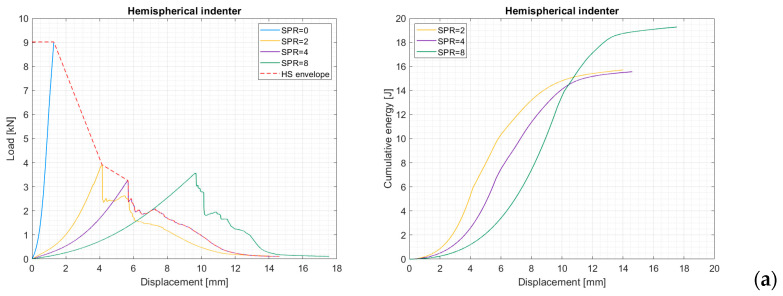

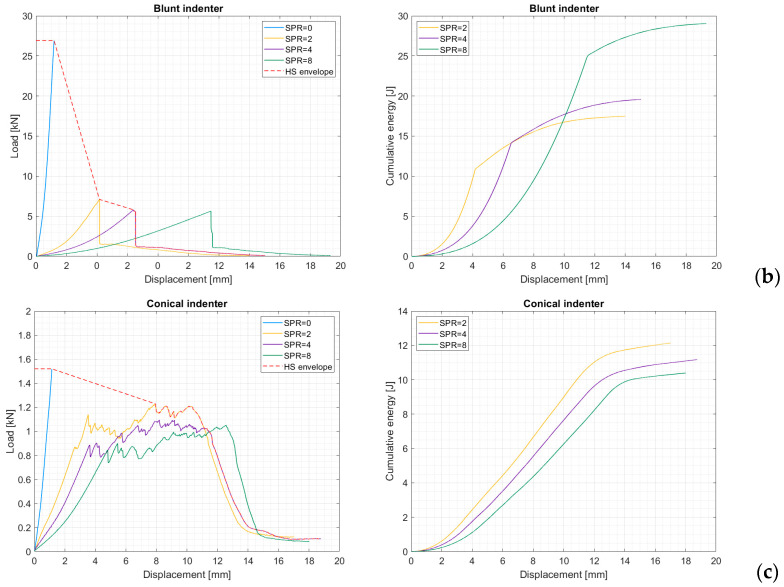
Force–displacement curves for continuous-fiber-reinforced composite plates and cumulative energy vs. displacements for (**a**) the hemispherical, (**b**) the blunt, and (**c**) the conical indenters.

Operatively, the hydrostatic envelope is obtained from the envelope of the force–displacement curves obtained with SPR values of 0, 2, and 4 (see Figure 4 and Figure 5). The corresponding energy is computed through integration of the hydrostatic envelope.

The resulting force–displacement diagrams presented in Figure 5 for different indenter shapes show a typical behavior that is characterized by an initial smooth increase in the force, which, after reaching a peak value, drops considerably, thus suggesting the fiber failure initiation. Depending on the indenter’s shape, the failure can be sudden and almost total, as in the case of the blunt indenter, while, as in the case of the hemispherical and conical indenters, the plate failure is more gradual. Indeed, some successive, smaller peaks can be identified after the main failure. Interestingly, in the case of the conical indenter, after the first peak, the force required to pierce through the plate increases up to the complete failure of the reinforcing fibers. This may be caused by the fact that after cutting through the first layers of fibers thanks to the highly concentrated load, the conical indenter must enlarge the hole against the resistance of the surrounding fibers. Similar behavior is shown by the hemispherical indenter, but this effect is less evident, as most of the fibers tend to fail in a block. Therefore, it is observed that the behavior of the hemispherical indenter lays in the middle between the blunt and the conical ones. Regarding the diagrams for SPR = 0, it is evident that the degree of load concentration plays a major role in determining the crushing resistance of the composite plate. Indeed, the sharper the indenter, the lower the load needed to crush the plate. Speaking of the absorbed energies at different SPR values, in general, the energy absorbed at SPR = 2 and SPR = 4 is comparable, while it is higher for SPR = 8. This is expected, because at higher SPR values, the plate becomes more compliant, and thus more energy can be absorbed before the initiation of the fiber failure. This last observation, however, is not true in the case of the conical indenter, and the hypothesized cause of this behavior is that the sharper indenter is so efficient in piercing through the first layers of fibers, and thus starting the failure process, that the increase in energy absorption due to increased compliance is inhibited.

### 2.5. Preliminary Dynamic FEM Analysis of Short-Fiber-Reinforced Plates

To check the reliability of the QSPM for short-fiber-reinforced plates, a simulation of the dynamic impact of a projectile onto the plate was set up. For this purpose, the Mechanical solver inside of the Ansys^®^ 2023 was used to perform an explicit dynamic simulation. The “active” part of the plate with SPR = 8 was modeled, which consists of a circular plate with a diameter of 102 mm. Moreover, only a fourth of the system was simulated through proper exploitation of the symmetries. The plate was fixed on its external sides, while the projectile’s movement was confined only in the perpendicular direction with respect to the plate. During the simulation, the projectile was left to impact the plate with a given initial velocity, and its kinetic energy was to be dissipated by the interaction with the plate. The projectile was modeled as a rigid body made of steel with mass of 19.84 g, while the plate’s mechanical behavior was modeled with isotropic elasticity and isotropic multilinear hardening. The choice of modeling the Smooth PA as an isotropic material (see Table 3) was justified by its highly plastic behavior shown in both tensile and indentation tests, thus suggesting a minor influence coming from the presence of the fibers. The data concerning the mechanical behavior of the plate were produced by means of proper tensile testing on 3D-printed specimens (Figure 4b).

**Figure 6:**
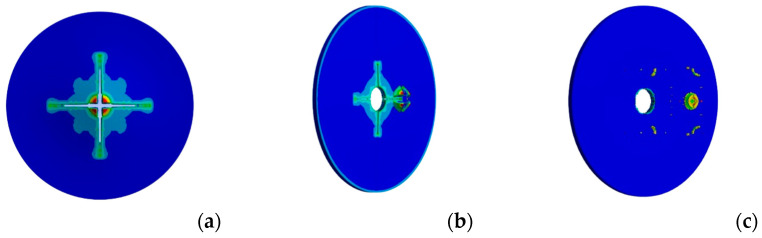
Fracture mode of the plate (contour showing EQPS) after penetration of the blunt projectile. Impact at (**a**) 25 m/s, (**b**) 40 m/s, and (**c**) 70 m/s. [Equivalent plastic strain 0 ÷ 0.035].

To determine the fracture locus, a maximum equivalent plastic-strain-based criterion was used [21]. Moreover, friction was also considered in the interaction between the projectile and the plate by setting the dynamic friction coefficient to 0.3 [22]. The resulting mesh of the system consisted of a structured grid made of 80,000 linear hexahedral elements, as shown in Figure 6.

## 3. Results and Discussion

### 3.1. Quasi-Static Punch Model Applied to Short-Fiber-Reinforced Plates

**Figure 7:**
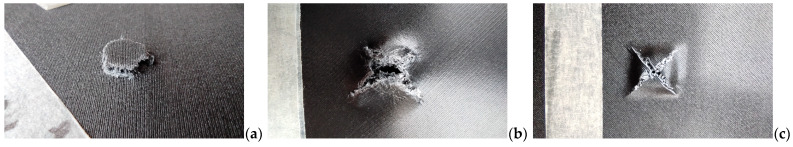
Area damaged with (**a**) blunt, (**b**) hemispherical, and (**c**) conical punches—short fibers.

Due to a lack of studies in the scientific literature involving the impact characterization of short-fiber-reinforced plates printed using the FFF method, it is non-trivial to assess the reliability of the quasi-static penetration model when applied to such components. Indeed, the model was explicitly validated by Gama and Gillespie [14] on continuous-fiber-reinforced composite thick plates penetrated by a blunt indenter (Figure 7). Moreover, comparison with the available literature is difficult not only because of different testing conditions, as low-velocity impacts are strongly affected by the boundary conditions (markedly, the SPR value), but also because the available studies try to assess the impact energy that allows for the piercing of the plate rather than trying to quantify the absorbed energy. However, it is still meaningful to try to compare the results of the QSPM and the FEM analysis produced in the present work with some available data from the literature; these results are conveniently summarized in Table 4. A reasonable comparison with the results obtained in the present study can be made considering the energy absorbed by the hemispherical indenter, which can better mimic the spherical impactor typically used in the tests found in the literature. By normalizing the results of the hemispherical projectile with respect to the plate’s areal density at SPR = 8, the energy absorbed by damaging the plate at the ballistic limit is estimated to be 10.5 J/(kg/m^2^). In general, the literature shows that the normalized energy of low-velocity impact that causes the complete perforation of a plate is found in the range of 0.5–4.4 J/(kg/m^2^). Therefore, the method of Gama and Gillespie seems to overestimate the energy that the plate can absorb. In addition to these experimental observations, the FEM model of the plate has also shown drastically lower impact energy at the ballistic limit. According to the numerical results, the ballistic limit for a hemispherical projectile with a mass of 19.84 g is around 16 m/s, which results in 2.54 J or 0.88 J/(kg/m^2^). The issue when dealing with short-fiber-reinforced plates is the considerable difficulty in precisely determining the onset of failure for the crushing test (SPR = 0) foreseen in the QSPM, because the curve is rather smooth and no sudden drop in load can be easily detected. Instead, only a slight change in the slope of the curve suggests plastic deformation. Moreover, the mechanical behavior shown by specimens printed with the short-fiber-reinforced filament suggests that the main damage mechanism is plastic deformation, and only a minor role is played by the reinforcement. It is also worth considering that quasi-static tests are not capable of capturing the effect of the strain rate, which can be expected to be quite influential for a thermoplastic-based composite; thus, the energy absorbed might also be overestimated for this reason. This last observation is an aspect that deserves further research.

### 3.2. Quasi-Static Punch Model Applied to Continuous-Fiber-Reinforced Plates

**Figure 8:**
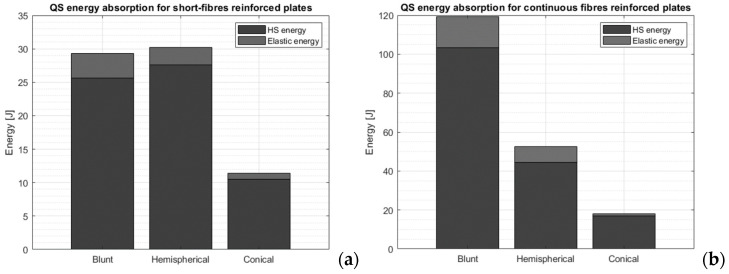
Energy absorbed by the plates reinforced with short (**a**) and continuous (**b**) fibers according to the quasi-static punch model.

According to the QSPM proposed by Gama and Gillespie, most of the energy absorbed by the plate is related to the damaging of the material, while only a minor part is associated with the elastic strain energy. The results for different indenters show that the nose shape of the indenter plays a major role in determining the energy the plate can absorb. The conical shape is the most efficient in penetrating the material, followed by the hemispherical and the blunt ones (see Figure 8).

**Figure 9:**
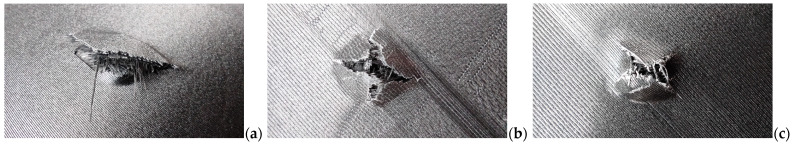
Area damaged with (**a**) blunt, (**b**) hemispherical, and (**c**) conical punches—continuous fibers.

In general, the sharper the indenter’s nose, the more concentrated the load, which leads to requiring lower peak loads to break through the first layers of the plate and start the damage (Figure 9). This is evident from the lower peak loads observed at SPR = 0 and beyond. These findings seem to be in contrast with the observations made by Gellert et al. [17] regarding traditionally manufactured glass-fiber-reinforced composite plates, as the scholars found that the energy absorbed by thin plates is not significantly influenced by the projectile shape during a ballistic impact. This may be due to the fact that 3D-printed plates are reinforced with a non-woven continuous filament, which is particularly weak with respect to penetrating loads, as shown by Cheng et al. [12]. As in case of the short- fiber-reinforced plates, the reliability of the QSPM suggested by Gama and Gillespie can be verified through a suitable comparison with experimental data available in the literature. The most appropriate test to be used for comparison is again the one involving the hemispherical indenter, as the tests available in the literature were conducted in similar conditions. As it can be seen in Table 5, the normalized energy absorbed by the plate is in good agreement with the literature, thus suggesting a satisfying reliability. However, it must be noticed that the studies present in the literature are very few and exhibit different system parameters, such as fiber volume fraction, SPR, and materials, which makes it difficult to irrefutably prove the reliability of the QSPM.

### 3.3. Plate Strengthening through Addition of Continuous Fibers

**Figure 10:**
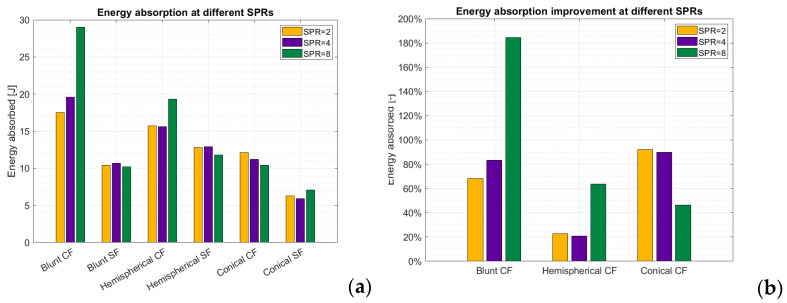
(**a**) Energy absorbed by the plates reinforced with continuous fibers according to the quasi-static punch model. (**b**) Percentage of improvement of energy absorbed with respect to short-fiber-reinforced plates when the continuous filament is added as reinforcement.

As it has been shown previously, the reliability of the QSPM test for short-fiber-reinforced plates () is insufficient for using those results as a comparison for the improvement of energy absorption capabilities of the plates when the continuous filament is added as reinforcement. Therefore, to weigh short-fiber-reinforced plates against continuous-fiber-reinforced ones, it is more meaningful to compare the single indentation tests at varying SPRs. As it can be seen in Figure 10, there is no doubt about the huge improvement that the continuous reinforcement brings to the plates in terms of energy absorbed.

No matter the shape of the indenter, the improvement is always higher than 20%, and it goes up to over 185% in the case of the blunt indenter for SPR = 8. Interestingly, while the cases with SPR equal to 2 and 4 show a similar improvement, the case with SPR = 8 is significantly higher for the blunt and hemispherical indenters and lower for the conical one.

### 3.4. Plate Strengthening through Addition of Continuous Fibers

Making a solid comparison between traditionally manufactured composite plates and FFF-printed plates is not trivial, mainly because of the high variability of the parameters involved in the different experimental procedures presented in the literature. However, we tried to understand how this novel composite printing technique ranks with respect to traditional manufacturing methods. The data retrieved from the literature for carbon–epoxy composite plates is illustrated in Table 6, along with the results of the QSPM adopted in the present study. Looking at the normalized impact energy, the data suggest that the FFF-printed plates reinforced with the continuous filament may behave even better when subjected to impact with respect to traditionally manufactured carbon–epoxy plates. In particular, the blunt indenter exhibits the highest ballistic performance difference, while the hemispherical one seems to be in range with the traditional composites, even if it is slightly more performant. On the other hand, the conical indenter shows a poorer ballistic performance compared to traditional composites, thus confirming the fact that the non-woven reinforcement is more sensitive to sharp penetrators piercing through adjacent paths. Nonetheless, these conclusions must be taken with the correct level of caution, as the QSPM was not directly validated by ballistic testing, but its reliability was assessed indirectly through comparison with the literature, which, due to a lack of specific studies, was possible only in the case of the hemispherical indenter.

### 3.5. Traditional vs. FFF-Printed Plates: Quasi-Static Loading

Comparing the performance of FFF-printed composite plates and traditionally manufactured ones is simpler in the case of quasi-static loads, because instead of relying on results produced by a model, it is possible to directly compare the experimental results, thus obtaining a more solid evaluation. The experimental results for quasi-static indentation tests obtained in the present work were compared with suitable studies available in the literature. As it can be seen from the comparison with the literature, it is undisputable that the FFF-printed composite plates possess a comparable quasi-static energy absorption capability with respect to traditionally manufactured composites. Nonetheless, the manufactured composite still behaves generally better than FFF-printed ones, mainly because of the higher fiber volume fraction achievable. Another interesting remark is that thermoplastic composite plates tend to have more comparable energy absorption capabilities with respect to FFF-printed ones, while the thermoset composites behave slightly better.

## 4. Discussion

### 4.1. Uncertainty in the Quasi-Static Punch Model

Due to a lack of direct experimental validation, it was not possible to comprehensively verify the reliability of the QSPM. Nonetheless, thanks to the comparison of the results with the available experimental data found in the literature and the FEM impact simulation, the QSPM was found to largely overestimate the absorption capabilities of short-fiber-reinforced plates. This, however, was expected, as Gama and Gillespie developed this model explicitly for thick composite plates reinforced with continuous fibers. Similarly, the QSPM was validated for the composite plates reinforced with the continuous carbon filament. In this case, the results achieved by means of the QSPM were in good agreement with the data found in the literature for FFF-printed composite plates. However, the limited number of prior impact studies involving FFF-printed composites did not allow for a comprehensive validation of the QSPM. Indeed, the experimental data that can be found in previous studies mainly involve impactors with a hemispherical nose shape or spherical projectiles. Moreover, the validation of the QSPM through comparison with the literature also suffers from the fact that impact tests involving FFF-printed composites are characterized by a huge variability in the test parameters, such as the materials, printing process, and impact velocity. Therefore, to ensure the complete validation of the QSPM for FFF-printed plates reinforced with continuous fibers, direct experimental investigation by means of ballistic testing is needed.

### 4.2. Performance Increase through Addition of Continuous Fibers

By considering the experimental data obtained in the present work regarding FFF-printed composite plates, it is possible to assess that the addition of the continuous carbon filament has a significant effect in the improvement of both the indentation resistance and the ballistic performance of the plates. Quantitatively speaking, the improvement in the energy absorbed by the plate against a penetrating static load ranges between 20% and 185%. As regards the energy absorbed during a dynamic load, instead, it is difficult to make a reliable quantitative evaluation of the performance improvement when adding the continuous fiber, mainly because the QSPM was revealed to be ineffective in estimating the energy absorption when applied to the short-fiber-reinforced plates. However, considering both the FEM results and the data from the literature for short-fiber-reinforced plates, it is possible to assert that the improvement in energy absorption could reasonably be one order of magnitude, i.e., from 10^0^ J/(kg/m^2^) to 10^1^ J/(kg/m^2^).

### 4.3. Overall Performance of FFF Plates Reinforced with Continuous Fibers

When it comes to comparing FFF-printed composite plates with traditionally manufactured ones, overall, FFF-printed plates suffer from the limited fiber volume fraction that can be achieved. Nonetheless, the results are promising. When it comes to impact performance, FFF-printed plates behave comparably to—if not better than—traditional composite plates. However, it must be taken into account that the ballistic results are only an estimation coming from the QSPM, which was validated partially due to the lack of impact studies involving FFF composite plates. That said, the static indentation tests also confirmed the potential of this novel AM technique for composite materials. The comparison between static indentation tests performed on FFF-printed plates and traditional ones indeed revealed a comparable energy absorption performance, despite presenting a much lower fiber volume fraction. Considering what has been said so far, it is evident that FFF-printed composites reinforced with continuous fibers can be competitive in terms of penetration resistance with respect to traditionally manufactured composites. This is even more impressive when considering that FFF-printed composites are characterized by lower values of fiber volume fraction, which means that it is possible to reach comparable strength levels with reduced consumption of reinforcing materials. However, the main issues affecting FFF printing of composites remain the high reinforcing material’s cost and the low production speed. On average, the CCF-1.5K pre-impregnated carbon filament used in the present study can be found online at an estimated cost of USD 3000/kg, while aerospace-grade carbon fiber is available at around USD 90/kg. Both of these issues, however, are somehow balanced by the design freedom inherent in the FFF process. Indeed, components that should not resist penetrating loads do not need necessarily to be printed with full infill, but instead the reinforcement could be laid precisely only where needed. The strength of this novel printing technique clearly lies in the possibility of producing optimized reinforcement with reduced weight and, thus, costs. Moreover, the prospect of obtaining a net shape after the print allows for reducing the costs of post-processing, while not eliminating them completely, as well as the risks of introducing defects in the composite part. For instance, the composite plates that were printed for the present study did not require any drilling operation for the perimetral holes, thus reducing the risk of degradation of the mechanical properties and the need for additional machinery.

## 5. Conclusions

In conclusion, the FFF printing technique for continuous-reinforced composites has been shown to be competitive with traditional manufacturing processes concerning the impact and static penetration loading conditions, even if it is characterized by a lower fiber volume fraction. However, the main limitations of this novel AM technique are the reinforcement cost and the low production speed, which limit the employment of this manufacturing process for large production batches. That said, the use of FFF printing for composites may be suitably implemented for small production batches with highly customized components, such as prostheses. Another fundamental advantage of this AM process is the relative ease of implementation, as the process requires neither supplementary expensive equipment nor particularly skilled operators to be proficiently used. These characteristics make FFF composite printing particularly suitable for small companies, such as start-ups, that may have a limited number of resources for the set-up of a small production system with high flexibility.

## Figures and Tables

**Table 1:** Properties of the materials.

Material	Properties *			
	Diameter [mm]	V_f_ [−]	UTS [MPa]	E [GPa]
Smooth PA	1.75	-	71.8	5.82
CFC PA	1.75	-	41	1.25
CCF-1.5K	0.36	0.6	2206	149

* V_f_: volume fraction; UTS: ultimate tensile strength; E: elastic modulus.

**Table 2:** Selected parameters.

Parameter	Selection
Print speed	10 mm/s
Layer thickness	0.36 mm
Hatch spacing	0.65 mm
Nozzle temperature	250 °C
Fiber layout	0°/90°

**Table 3:** Mechanical properties of tensile specimens made of Smooth PA.

Property	Value
Elastic modulus	3 GPa
Poisson’s ratio	0.4
Yield strength	30 MPa
Ultimate tensile strength (UTS)	50 MPa
Equivalent Plastic Strain at Failure (EQPS)	0.035

**Table 4:** Available results from the literature of ballistic impact tests on 3D-printed plates made of short-fiber-reinforced nylon.

Study	Test	Material	SPR	Impact Velocity [m/s]	Areal Density [kg/m^2^]	Impact Energy [J]	Normalized Impact Energy [J/(kg/m^2^)]
[23]	Ballistic	Nylon—short fibers	11.1	95	2.925	14	4.8
[24]	Ballistic	Nylon—short fibers	12.6	208	4.204	44.5	10.6
[25]	Ballistic	Nylon—short fibers	12.6	21	0.870	0.5	0.5
[25]	Drop test	Nylon—short fibers	5.1	2.7	0.870	5.0	4.4
[26]	Drop test	Nylon—short fibers	3.0	4	3.000	8.7	2.9
FEM	N/A	Smooth PA	8.0	16	3.150	2.54	0.9
QSPM	Quasi-static	Smooth PA	8.0	N/A	2.880	30.2	10.5

**Table 5:** Results available from the literature regarding ballistic impact tests on 3D-printed plates reinforced with continuous fibers.

Study	Test	Material	V_f_ [−]	SPR	Impact Velocity [m/s]	Areal Density [kg/m^2^]	Impact Energy [J]	Normalized Impact Energy [J/(kg/m^2^)]
[6]	Drop test	Nylon and glass fibers	0.32	4.7	3.88	4.09	99.2	24.6
[26]	Drop test	Nylon and glass fibers	0.54	3.0	4	3.000	79.2	23.3
QSPM	Quasi-static	Reinforced nylon and carbon fibers	0.23	8.0	N/A	2.909	52.6	18.1

**Table 6:** Energy absorption results concerning ballistics tests on traditional composite carbon–epoxy plates and QSPM on FFF.

Study	Test	Projectile Shape	SPR	Impact Velocity [m/s]	Areal Density [kg/m^2^]	Impact Energy [J]	Normalized Impact Energy [J/(kg/m^2^)]
[27]	Ballistic	Blunt	8.0	82	3.660	47	12.8
[27]	Ballistic	Hemis.	8.0	79	3.660	44	12.0
[27]	Ballistic	Conical	8.0	83	3.660	48	13.1
[15]	Ballistic	Blunt	8.0	80	6.355	96	15.0
[28]	Ballistic	Blunt	N/A	114	5.250	87	16.6
[29]	Ballistic	Conical	N/A	44	3.506	29	8.3
QSPM	Quasi-static	Blunt	8.0	N/A	2.909	119.4	41.0
QSPM	Quasi-static	Hemis.	8.0	N/A	2.909	52.6	18.1
QSPM	Quasi-static	Conical	8.0	N/A	2.909	18.2	6.3
